# Milstein Medical Asian American Partnership (MMAAP) Foundation invites applications for 2020 Irma and Paul Milstein Program for Senior Health Fellowship and Research Project Awards in Geriatric Medicine and Aging Research

**DOI:** 10.1002/agm2.12084

**Published:** 2019-10-22

**Authors:** Sean X. Leng

**Affiliations:** ^1^ Milstein Medical Asian American Partnership Foundation New York NY USA

## 1 INTRODUCTION OF MILSTEIN MEDICAL ASIAN AMERICAN PARTNERSHIP FOUNDATION

Under the vision and leadership of Founder and Chairman Howard P. Milstein, Milstein Medical Asian American Partnership (MMAAP) Foundation was founded in 2011 to improve world health by developing mutually beneficial partnerships between the US and China, as well as greater Asia. Working with some of the world's premier health organizations, MMAAP Foundation's priority is to bring together and fund exchanges among the best medical talents and institutions in the regions.

For more than 50 years, the Milstein family has been actively involved in health and medical‐related philanthropy. MMAAP Foundation builds upon this distinguished history in areas that include geriatric medicine (under Irma and Paul Milstein Program for Senior Health), dermatology, translational medicine, and stem cell research and regenerative medicine.

With the generous support from Mr. Milstein, MMAAP Foundation has contributed nearly $4.5 million in funding thus far. It has awarded over 60 Fellowship and Research Project Awards to support the work of exceptional Chinese physicians, scientists, and investigators demonstrating vision, drive, and dedication to making discoveries and advancements in these important medical fields. The Foundation has partnered with over 30 prominent US institutions, including Harvard University and Johns Hopkins University, and over 30 Chinese institutions, including the Peking Union Medical College Hospital and the Chinese Academy of Medical Sciences. Over 70 scientific publications have been published in leading English and Chinese professional journals with acknowledgment of the Foundation's support.

In 2018, MMAAP Foundation successfully registered its China Representative Office in Beijing. The Foundation is one of the few nongovernmental organizations that have obtained an official presence in China. This strengthens its philanthropic efforts in China and further promotes scholarly exchanges and the bilateral relationship between China and the US.

MMAAP Foundation has become a highly respected international foundation demonstrating expertise in producing medical and scientific progress with integrity and efficiency.

For more information, please visit MMAAP Foundation bilingual website (English and Chinese/Mandarin) at <http://www.mmaapf.org>. MMAAP Foundation is a 501(c) (3) non‐profit organization.

## 2 2020 MMAAP FOUNDATION FELLOWSHIP AND RESEARCH PROJECT AWARDS

### 2.1 Applications invited

MMAAP Foundation invites the submission of applications for Irma and Paul Milstein Program for Senior Health Fellowship and Research Project Awards. The aim of the program is to build enduring partnerships between the US and China, as well as greater Asia, through the training of future Chinese academic leaders and the funding of research projects that will have potential for immediate impact on improving geriatrics practices and senior health in China and around the world.

### 2.2 Award details and eligibility

Irma and Paul Milstein Program for Senior Health Fellowship Award supports an applicant to have 1 year of training at a prominent sponsoring institution in the US. The award will provide $85,000, including $60,000 to cover the fellow's expenses and $25,000 to cover research expenses at the US sponsoring institution. Applicants should: demonstrate excellence in geriatric medicine and gerontology; be at the rank of instructor or above with an MD or PhD; have significant research experience with a distinguished publication record; have commitment to research; demonstrate leadership skills and excellent English proficiency; and have the agreement of a US institution to provide his/her training.

Irma and Paul Milstein Program for Senior Health Research Project Award supports a new or ongoing clinical research project with demonstrated potential for immediate impact on improving senior health in China with the amount of $60,000. The award will provide $50,000 to the Chinese home institution where the research is to be conducted, and $10,000 to the US partner institution. The applicant must serve as the principal investigator (PI) of the proposed project with a representative from a prominent US institution as partner, who will serve as the co‐PI. Both Chinese and US leaders must have demonstrated productivity as clinical investigators. The successful application should include a stated goal of establishing a partnership between the US and Chinese institutions to facilitate a long‐term relationship and a feasible plan for continuing collaboration.

Note: Applicants should contact the US sponsoring/partner institution to determine if the sponsoring/partner laboratory receives funding from the National Institutes of Health (NIH) and, if so, whether there are established policies to address the relationship between NIH‐funded research and the research proposed in the application being submitted. No indirect cost will be allowed. The applicant cannot use the same research proposal to apply for both the Fellowship and Research Project Awards.

### 2.3 Contents of applications

All applications should be prepared in one PDF document with contents in the following order. The PDF should contain both Chinese and English versions:

1. *Title page*, including applicant's name, title of proposal, Chinese home institution, and US sponsoring/partner institution. *Title page* and *checklist* (in both Chinese and English) can be downloaded from http://www.mmaapf.org.
2. *A letter from the applicant stating*:
  - research goals and plan for future collaboration with the US sponsor/partner institution
  - clinical and translational impact of the project
  - description of how this project will yield measurable results over a 2‐year time period
  - acknowledgment of the requirement to submit periodic progress reports every 6 months and an expenditure report at the end of the grant period
  - acknowledgment of the requirement to list *Irma and Paul Milstein Program for Senior Health, MMAAP Foundation* support in all presentations and publications
  - acknowledgment to keep MMAAP Foundation informed of current/future presentations and/or publications.
3. *Curriculum vitae of the applicant* (up to six pages), including date of birth, education and training, academic appointments, key publications, and other research support.
4. *A recommendation letter* from the President of the applicant's home institution or the Chair of the applicant's home department. This letter must evaluate: (a) the quality of previous and proposed research; (b) commitment of the applicant and his/her department and institution to clinical investigation; (c) the applicant's ability to understand, write, and speak English; (d) the provision of sufficient time to achieve the research goals proposed in the application or for the fellow upon return to China to resume research within 6 months; (e) guaranteed access to facilities and support allowing the applicant to conduct the research or continue the research upon returning to China (this includes provision of consumable supplies and access to necessary equipment); (f) the applicant's willingness to continue the research project after his/her return to China and the collaboration with the US sponsoring institution (for Fellowship Award); and (g) a guaranteed faculty position at the applicant's home institution upon returning to China (for Fellowship Award).
5. *A support letter from the mentor/co‐PI at the US sponsoring/partner institution* addressing the relationship between NIH‐funded research, if any, and the proposed research in this application and accepting the applicant to work on the research detailed in the application (for Fellowship Award) or acknowledging the co‐PI's willingness to assist in planning and implementation of the proposed research (for Research Project Award). If the applicant cannot identify a US sponsoring/partner institution, she/he is advised to review the list of senior investigators and their research areas on the MMAAP Foundation's website under the “US Experts” tab and directly contact them to seek collaboration, if appropriate.
6. *A curriculum vitae of the mentor/co‐PI at the US sponsoring/partner institution*.
7. *A research proposal* (3‐5 pages, including references) prepared by the applicant detailing the title, aims, significance, approach, and projected results. The proposal must be typed in *Arial 11‐pt. type with 1‐inch margins*. Note: Human and/or animal use must be approved by the Institutional Review Board or equivalent and documented before funding begins.
8. A line‐item *budget* and justification for the research project for both Chinese and US institutions must be attached (for Research Project Award only). Matching funds by the requesting partner institutions, if necessary, are encouraged (for Research Project Award only).

Deadline for receipt of applications is on Monday, February 3, 2020 at 11:59 pm (China time). Decisions will be announced with funding available in mid 2020. Incomplete applications will not be considered. Applications need to be uploaded to MMAAP Foundation website at <http://www.mmaapf.org>. Please contact info@mmaapf.org or call +1 (212) 850‐4505 with any questions.

### 2.4 Peer‐review process

Applications (Chinese version) will first be reviewed by Chinese experts in geriatrics. The selected top applications (English version) will then be reviewed by the US experts in geriatrics. One fellowship award application and one research project award application with the highest quality will be selected through this two‐step peer‐review process with final approval by MMAAP Foundation.

